# Red Cabbage: A Novel Treatment for Periductal Lactational Mastitis

**DOI:** 10.7759/cureus.33191

**Published:** 2022-12-31

**Authors:** Chiya Abramowitz, Aryeh Deutch, Ethan Shamsian, Yakubmiyer Musheyev, Farage Ftiha, Shira Burnstein

**Affiliations:** 1 Internal Medicine, New York Institute of Technology College of Osteopathic Medicine (NYITCOM), Glen Head, USA; 2 Medicine, New York Institute of Technology College of Osteopathic Medicine (NYITCOM), Glen Head, USA; 3 Medicine, New York Institute of Technology College of Osteopathic Medicine (NYITCOM), Old Westbury, USA; 4 Internal Medicine, Maimonides Medical Center, Brooklyn, USA

**Keywords:** breast abscess, mastitis, cabbage, breastfeeding, lactational mastitis

## Abstract

Lactational mastitis is a common condition in breastfeeding women that manifests as painful, swollen, and erythematous breasts. Customary treatment guidelines include antibiotics, cold compression, and continued breastfeeding. This report highlights a unique case in which the symptomatic progression of lactational mastitis was alleviated with the utilization of red cabbage and warm compresses. The study further investigates the potential benefits and role of red cabbage in treating mastitis infections to prevent unnecessary overuse of antibiotics and promote antibiotic stewardship practices.

## Introduction

Breast engorgement and mastitis are painful conditions that affect postpartum women. Mastitis is a condition defined by the inflammation of the breast tissue and can be infectious or non-infectious in etiology. It consists of varying subtypes, including puerperal mastitis, otherwise known as lactational mastitis, and nonpuerperal mastitis, which includes periductal and granulomatous mastitis [1].

Lactational mastitis is a relatively common condition in breastfeeding women and can affect up to 33% of lactating women [2]. It most often occurs in the first three months of breastfeeding but may also occur throughout the entire breastfeeding period [3]. In this condition, the breast becomes erythematous, swollen, and painful due to an abundance of milk production along with simultaneous poor milk drainage, both of which are associated with milk stasis and engorgement. Poor milk drainage can result from structural abnormalities (e.g., partial blockage or swelling of milk ducts and nipple cracks, excoriation, or trauma), overproduction of milk (e.g., infrequent feedings, rapid weaning, maternal malnutrition, illness, or fatigue) or other factors (e.g., use of topical antifungals and chronic recurrent mastitis) [4,5]. The resulting stasis can lead to inflammatory pathologies in the breast tissue, such as non-infectious mastitis or galactophoritis, as well as secondary bacterial mastitis [1].

The severity of mastitis may vary from self-limiting and non-infectious inflammation to severe, abscess-forming infections. The diagnosis is commonly made based on anamnesis and is clinical in nature [5]. Patients typically present with unilateral erythema, swelling, and tenderness in either the partial or the complete breast area [4]. If symptoms persist for over 12-24 hours, bacteria may accumulate in the stagnant breast milk, leading to the development of infective lactational mastitis. This condition presents with firmness, tenderness, and redness of the breast as well as flu-like symptoms such as fever, malaise, myalgias, chills, and in some cases, reactive axillary lymphadenopathy. In the early stages, a physical examination may only indicate a few clinical signs, such as cracks and sores on the nipple or purulent papillary secretion without systemic symptoms. Cultures of breast milk and blood may confirm the causative agent but are only warranted in the presentation of severe symptoms or sepsis. Ultrasound and other imaging techniques may be used when differentiating breast abscesses from mastitis. If malignant breast disease is suspected and no improvement occurs after a few days of treatment, the patient should be referred for clinical mammography and biopsy [6].

Lactational mastitis is most commonly caused by Staphylococcus aureus and other penicillinase-producing staphylococci, with less frequent infections caused by other skin flora such as Streptococcus pyogenes, Bacteroides, Escherichia coli, and Corynebacterium [5]. However, methicillin-resistant staphylococcus aureus (MRSA) has risen as a more prevalent and more lethal agent of lactational mastitis [7]. For this reason, outpatient treatment of uncomplicated lactational mastitis includes either dicloxacillin, cephalexin, erythromycin, or clindamycin for those with penicillin hypersensitivity [8]. In patients with a high risk for MRSA, treatment can include clindamycin or trimethoprim-sulfamethoxazole for women breastfeeding full-term healthy infants more than one month old. In settings of severe infection, empiric inpatient treatment with vancomycin and subsequent targeted therapy is given. Incision and drainage of the pus may also be required in severe cases. Although the optimal duration of treatment is not established for uncomplicated lactational mastitis, five to seven days of treatment is usually sufficient if the response is rapid and complete, while a 10-14-day regimen has been shown to reduce the risk of recurrence. In addition to antibiotics, initial treatment of mild to moderate lactational mastitis may include nonsteroidal inflammatory agents and cold compresses to reduce swelling and pain, as well as continued breastfeeding to complete emptying of the breast. Frequent and complete breastfeeding as well as optimized breastfeeding techniques are also encouraged throughout the treatment duration as they can reduce the risk of developing recurrent and relapsing lactational mastitis [2].

Patients who are noncompliant with a pharmacological approach to treating mastitis may benefit from an adjunctive topical use of cabbage to alleviate the symptoms. There have been numerous articles written in medical blogs and self-help medical websites such as Healthline [9] and WebMD [10] that advocate for the topical use of cabbage to treat the symptoms of mastitis. To date, the literature on the utility of cabbage for treating mastitis has mainly focused on the effect of cabbage on breast engorgement, one of the main causes of mastitis [11], though not much is known about its mechanism of action. In larger studies, cabbage has been demonstrated to alleviate breast pain and the hardness of the engorged breasts, as well as increase the length of breastfeeding throughout the course of the infection [11,12]. In addition, while warm compresses have been documented to drain clogged milk ducts [13], there may be potential benefits when utilizing cabbage in conjunction with warm or cold compresses [14]. The use of cabbage in place of antibiotics in treating lactational mastitis may also have additional potential benefits, such as reducing the time to systemic infection and decreasing the possibility of creating more antibiotic-resistant bacterial strains. Here, we present a case of a patient presenting with recurrent lactational mastitis that resolved with the use of red cabbage leaves instead of the traditional antibiotic regimen for treating mastitis.

## Case presentation

A 28-year-old Caucasian gravida 2, para 2 (G2P2) female who had been breastfeeding for six months presented to her primary care physician with a three-day history of worsening right localized breast tenderness, swelling, and erythema. At the time, the patient was in her sixth month of breastfeeding her second child who was delivered six months prior to symptom onset. The breast tenderness was experienced both with and without breastfeeding and was exacerbated with physical contact with the affected breast. The erythematous region increased in size within a few hours of the onset of symptoms and slowly expanded for the following two days. The patient also noted red streaking erythema radiating toward the ipsilateral axilla approximately six hours before the office visit. She reported breastfeeding less frequently starting two days prior to the onset of symptoms as she desired to introduce supplemented milk formula and taper breastfeeding for her infant. She had first consulted an urgent care clinic and was prescribed antibiotic treatment but was non-compliant with medication adherence.

Her previous pregnancy was uneventful and was able to breastfeed her first child adequately without any difficulties or complications. She did not undergo any past breast surgeries and denied any past medical history of tuberculosis, sarcoidosis or any other infectious or granulomatous disease, Family history consisted of no known breast cancer or any notable breast diseases. Her only current medications consisted of a prenatal multivitamin, which she also took after her previous pregnancies.

**Figure 1:**
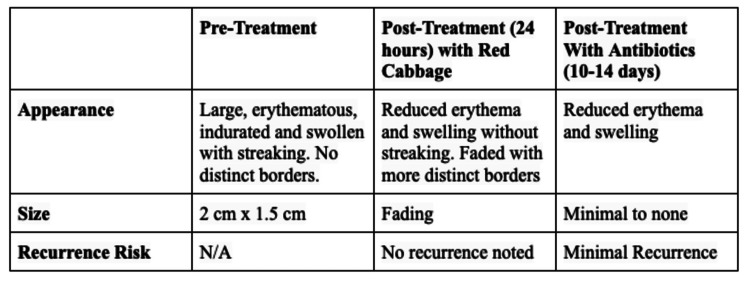
Pre-treatment and post-treatment effects of red cabbage on lactational mastitis infection

The patient was afebrile and normotensive on initial examination. Localized examination revealed an erythematous, tender, firm, irregularly shaped patch without distinct borders measuring 2 cm x 1.5 cm (Figure 1). The erythematous patch was located in the upper mid-lateral portion of the right breast, originating at the areola and centrifugally spreading to the region between the 9’ o'clock and 12 o’clock positions of the breast. Axillary lymph nodes were not palpable. She did not undergo mammograms or ultrasound imaging. Serum tumor markers were not measured.

The final diagnosis was lactational mastitis secondary to decreased breastfeeding, and the patient was informed that there was no malignancy. Due to the patient’s non-compliance, the patient was counseled to place a warm compress over a layer of a few outer wrapper red cabbage leaves directly covering the affected erythematous skin region for the entire duration of breastfeeding. The placement of the cabbage and warm compress was exclusively performed during all subsequent feedings. Within 24 hours of the initial topical cabbage treatment, regressions of erythema and tenderness were noted and the inflammation became more localized. An ultimate resolution of symptoms occurred 36 hours following the initiation of the topical cabbage treatment. When the patient followed up two days later, no erythema was noted, and the breast tenderness completely resolved.

## Discussion

Lactational mastitis is a complex condition whose pathophysiology involves poorly understood interactions between mammary-associated microorganisms and genetic factors of the host. Although current guidelines for the treatment of lactational mastitis include a regimen of antibiotics for febrile illness and symptomatic therapy, data on its treatment are limited. Emptying the breast and administering antibiotics may increase favorable clinical outcomes and significantly reduce the duration of symptoms [15]. Despite this, in more severe cases, these advantages are often negated by the additional incisions and drainage of pus that are indicated under the current clinical guidelines. These additional measures are often associated with prolonged healing time, regular dressings, difficulty in breastfeeding, and an increased risk of developing a milk fistula with unsatisfactory cosmetic outcomes [13].

In the patient presented in this study, the application of cabbage leaves on the affected area markedly decreased the length of illness and the need for antibiotic use. Since the patient did not take any other medications other than postnatal vitamins, no confounding effects from drug interactions influenced the results of red cabbage treatment.

The expanded use of red cabbage in treating lactational mastitis may have broader implications for antibiotic stewardship practices on a national level in the US. According to the Centers for Disease Control (CDC), approximately 125 million prescriptions for antibiotics, or 747 prescriptions per 10,000 people were issued in 2020 for female patients in the US, with 28% of annual antibiotic prescriptions being deemed unnecessary [16]. The most prescribed antibiotic was amoxicillin (119 per 10,000 people), followed by Azithromycin (84 per 10,000 people) and amoxicillin-clavulanate (64 per 10,000 people). Other commonly prescribed medications were cephalexin and doxycycline. Lactational mastitis has been estimated to occur in 2%-10% of breastfeeding women [17]. Given that affected patients often require a drug from the penicillin or cephalosporin class, the treatment of mastitis can contribute to the statistics above, particularly the percentage of antibiotics that are prescribed unnecessarily. Therefore, the development of a treatment protocol that lessens the need for antibiotics is desirable for the promotion of favorable antibiotic stewardship practices. Employing the use of red cabbage leaves in conjunction with other treatment guidelines can foster these desirable practices and improve patient outcomes for quicker illness resolution. 

Furthermore, the incidence of mastitis requiring hospitalization is low; in one cohort of 136,459 mothers, 127 women were hospitalized for mastitis, an incidence of nine per 10,000 deliveries [3]. Since lactational mastitis rarely progresses to severe disease, outpatient treatment with red cabbage may be beneficial.

The use of cabbage as an anti-inflammatory agent for conditions such as lactational mastitis is not a completely novel idea. It has been established in the published literature that cabbage contains increased levels of various substances that exhibit antioxidant properties such as polyphenols and flavonoids, which include anthocyanins, luteolin, myricetin, and quercetin [1,18]. These bioactive substances found in cabbage may serve as a direct link to the underlying reason for the tremendous potential of cabbage in treating peptic ulcers, gastritis, irritable bowel syndrome, liver cirrhosis, hepatitis, cancer, hypocholesterolemia, and pancreatitis [18].

Oral cabbage ingestion has been found to also help mitigate the detrimental effects of cardiomyocyte damage due to oxidative stress. That is, oxidative stress is the production of reactive oxygen species (ROS) due to mitochondrial dysfunction [18]. A study conducted in 2018 provided evidence that cabbage extract protects against oxidative stress in cardiac tissue by inhibiting ROS production and apoptosis and by preserving mitochondrial functions [18]. Furthermore, it has been observed that the anti-inflammatory activity of various types of cabbage differs within different growing districts and varies when cultivated using different conditions or subjected to different post-harvest treatments [19]. With the ever-expanding research of the scientific community, it is possible that the most potent form of anti-inflammatory cabbage has yet to be discovered. By virtue of this, as well as the lack of awareness among the larger medical community, it is evident that more research can be performed in this space.

While the literature has indicated the effectiveness of cabbage in reducing pain and hardness of breast engorgement and lengthening breastfeeding duration, this case indicates that the cabbage may also deter the onset of more severe symptoms such as fever and flu-like symptoms and prevent the transition from localized to systemic infection within 24 hours. This case also indicates that red cabbage treatment may be used in conjunction with hot compresses, a known symptom reliever, to prevent progression to systemic mastitis infection and lead to the ultimate resolution of symptoms over a shorter period. Moreover, as seen in the case, treatment with red cabbage, rather than white or green cabbage, was shown to express a successful clinical outcome. Medical self-help websites such as WebMD encourage the use of white or green cabbage. However, there is scarce research supporting the use of green or white cabbage over red cabbage and more research needs to be conducted to ascertain the differences between the two types of cabbage.

Despite its potential, there are some uncertainties pertaining to the use of cabbage in treating lactational mastitis. For example, the application of organic foods such as cabbage may introduce various bacteria or fungi into the skin of the host, which may exacerbate the mastitis infection. The predominant organisms isolated from the outer wrapper leaves of freshly harvested cabbage leaves may include bacteria, yeasts, Alternaria spp., Aureobasidium pullulans, Botrytis cinerea, Cladosporium spp. and Penicillium spp. [20].

While all the beneficial effects of cabbage are still being uncovered and published for the wider community of healthcare professionals, more exploration is needed in order to ascertain the extent to which medical professionals can use a natural anti-inflammatory agent as opposed to the accepted first-line pharmaceutical agents. While cabbage is extremely effective, the production of greater side effects is yet to be known. For this reason, more research should be performed to achieve homogeneity across studies by using standardized assessments, such as duration of breastfeeding or pain engorgement scores, in order to objectively evaluate the efficacy of cabbage leaf treatment for lactational mastitis.

## Conclusions

In this case presentation, topical cabbage application was successfully used to alleviate the symptoms caused by lactational mastitis in an actively breastfeeding woman. The patient experienced less severe symptoms as the cabbage decreased the length of her illness and reduced her need for antibiotics. Not only would future patients benefit from these symptom-based advantages, but the medical community would benefit at large due to the attenuation of the challenge of antibiotic resistance. More research should be conducted in this field so that the mechanism through which the cabbage exerts its effects can be better understood, and that the usage of the product can be thoroughly refined.
